# Gamification and Serious Games in Orthopedic Education: A Systematic Review

**DOI:** 10.7759/cureus.68234

**Published:** 2024-08-30

**Authors:** Wei Shao Tung, Riley Baker, Kendal Toy, Mani Eftekhari, George Casey, Rana Jahani, Christopher Bono, Casper Harteveld, Lorena Bejarano-Pineda, Soheil Ashkani-Esfahani

**Affiliations:** 1 Department of Orthopaedics, Massachusetts General Hospital, Harvard Medical School, Boston, USA; 2 Foot and Ankle Research and Innovation Laboratory, Massachusetts General Hospital, Harvard Medical School, Boston, USA; 3 College of Arts, Media, and Design, Northeastern University, Boston, USA; 4 Department of Orthopaedic Surgery, Massachusetts General Hospital, Harvard Medical School, Boston, USA

**Keywords:** orthopedic surgery residency, orthopedic skills, virtual reality, gamification, serious gaming

## Abstract

Gamification and serious games have successfully been used in surgical specialties to improve technical skills related to systematic procedures. However, the use of gamified education material has remained limited in orthopedic residency training. The objective of this systematic review is to summarize the current use, development, and future directions of gamification for developing orthopedic skills.

A comprehensive literature search was performed on Ovid MEDLINE, Web of Science, and Scopus between January 1, 2012, and the search date of July 1, 2023. After screening 1,915 papers, a total of four publications that utilized elements of gamification in acquiring and/or improving orthopedic skills were included.

Three studies showed a positive correlation between video gaming experience and orthopedic skill performance, acquisition, or both. One study showed a positive response from residents when training sessions were hosted in a competitive, but friendly environment with direct observation from their attendings.

Gamified learning has the potential to improve orthopedic education, but its current use is largely unexplored. A competitive or rewarding environment promotes engagement and active learning. To enable the highest and most efficient level of training, future development should be geared toward virtual reality simulators that incorporate haptic feedback to better simulate other orthopedic-based tasks.

## Introduction and background

The newest generations of students entering medical school and graduate medical education are moving away from traditional textbook learning, with a much higher reliance on technology than any other generation of students [1]. To accommodate this trend, companies have developed software programs and games in an attempt to change the face of medical education [2,3] These applications made for medical education generally fall into two categories: education-based products with gaming features (“gamification”) and gaming-based products with an educational purpose (“serious games”). Nonetheless, current studies highlight that products on either end of the spectrum can stimulate active learning and promote motivation and engagement [4-7].

The general definition of gamification is the use of gaming mechanics in a non-gaming context [8,9] Examples include using leaderboards, quests, and narratives to assist in teaching and developing learning materials [1,8,9]. In serious games, students are immersed in a game environment to complete challenges that incorporate learning objectives [10]. This pedagogical approach can potentially emulate clinical scenarios where physicians think critically to help their patients [7-9,11]. Furthermore, such games allow students to build confidence through trial and error without real-world consequences, garner skills useful in professional settings, and progress up the learning curve at their own pace [7,12] They also have the potential to make improvements in medical education as they foster clinical problem-solving skills and can increase the efficiency of learning compared to other traditional medical education methods [13].

Extant studies have shown that games played on a variety of platforms such as consoles, smartphones, and virtual reality systems have been successful in reducing stress and anxiety while simultaneously proving helpful with knowledge acquisition [13-16]. Gamification and serious games can give frequent and immediate feedback to players on their performance, knowledge, or both without needing direct feedback from mentors or physicians. In addition, games are a more convenient and cost-effective alternative to other teaching methods, allowing access and exposure to educational materials at home, during downtime, or even on the go [2]. Integrating gamification, serious gaming, or both, into residency curriculums presents an opportunity to provide residents with an alternative way of alleviating stress while adequately preparing them for practice.

Current literature and anecdotal testimonies have illuminated significant gaps in orthopedic medical education [17,18]. Miyamoto et al. found no strong correlation between improved Orthopaedic In-Training Exam scores and the use of textbooks, review books, self-constructed flashcards, or lecture notes; however, they did find the use of prior exams and other question sources resulted in higher scores, a process more akin to gamification [18]. Therefore, there is a suggested need for the development of a new pedagogical approach in orthopedic education, whether it be clinical knowledge or skills. While the expansion of gamification for laparoscopic and endoscopic skills training in general surgery residency is plentiful, there is minimal research on this topic within the realm of orthopedics [6,19-21] As similar proficiency levels in dexterity, coordination, and visual-spatial skills are required for all laparoscopic, endoscopic, and arthroscopic procedures, there presents an opportunity to incorporate gamification and serious gaming across fields partaking in minimally invasive surgery (MIS) or more [22] Acknowledging the need to improve the orthopedic surgery education system, this systematic review aims to evaluate the current utility of gamified learning in orthopedic residency training programs and identify the opportunities for integration in the future.

## Review

Methodology

Development of Research Questions

As the aim of the study was to provide a detailed overview of the role of gamification and serious games in orthopedic education, a systematic review was chosen to best align with our objectives. This study focuses on the application of games in orthopedics, and the main concept of interest is the current state of orthopedic gamification, development, and future direction.

Relevant Studies

A comprehensive literature search was performed on Ovid MEDLINE, Web of Science, and Scopus ranging between January 1, 2012, and the search date of July 1, 2023, using the search strategy outlined in Appendix 1. The search dates were chosen to better reflect technological trends and the applicability of our findings.

Eligibility Criteria

All reviewed articles were published in English or had a peer-reviewed English translation available. Studies that indicated the use of entertainment technologies, gamification, and serious games in orthopedic training were considered. Aside from the exclusion of systematic reviews or meta-analyses, erratum or corrections, case reports, letters to the editor, or commentary articles, there were no other restrictions on study type. Other exclusion criteria included studies on non-orthopedic robotic surgical skills or studies with interventions that do not fully meet the gamification or serious gaming criteria, such as studies only indicating the use of a simulator or virtual reality without the incorporation of gamified elements.

Screening and Study Selection

**Figure 1:**
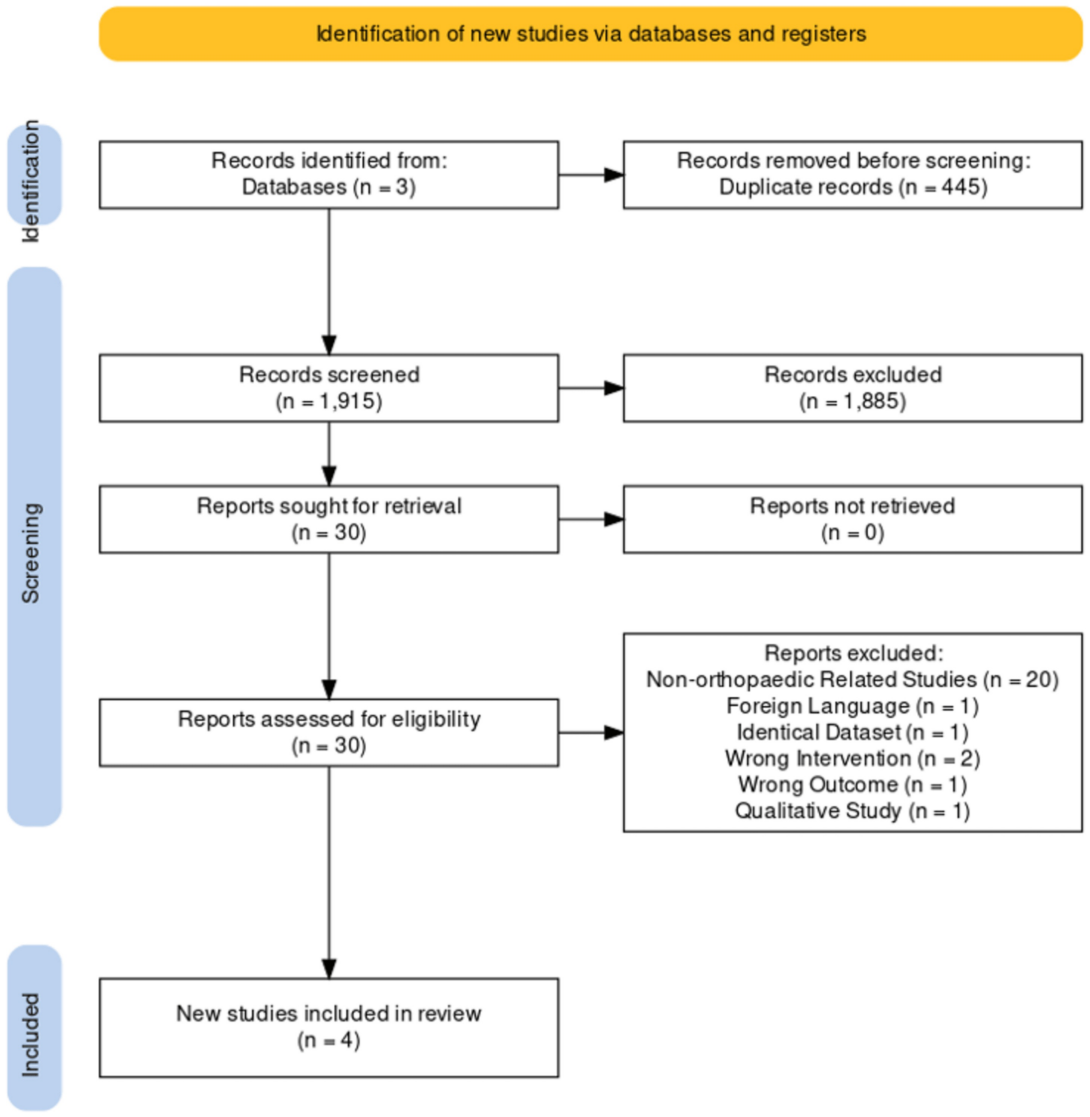
Preferred Reporting Items for Systematic Reviews and Meta-Analyses (PRISMA) flow diagram.

Study selection was performed in two stages by two reviewers (ME, GC). Screening of titles and abstracts was completed on a systematic review management platform, Covidence (Covidence, Melbourne, Victoria, Australia), which follows the Preferred Reporting Items for Systematic Reviews and Meta-Analyses (PRISMA) guidelines (Figure 1) [22]. Studies that met the initial eligibility criteria were isolated and a subsequent full-text review was conducted to screen for full inclusion. Disagreements on inclusion were resolved through discussion with a third reviewer (WST).

Data Extraction

A standardized data extraction sheet was made, which included the following study characteristics: author(s), year, country, study population and sample size, intervention, and results. Two reviewers (ME, GC) independently extracted and compiled the data into the prepared data extraction sheet. Another reviewer (WST) evaluated the accuracy of all data items collected for this study.

Results

In this study, a total of 2,360 studies were reviewed, with 1,915 articles remaining after the removal of duplicates. After title and abstract screening, 1,885 citations were removed based on exclusion criteria and 30 citations underwent full-text screening. Continuing with the screening process, 26 further studies were excluded, and four articles were included in the final review [23-28].

Study Characteristics

Across the four included studies (Table 1), 212 subjects participated in research to determine the effects of gamification and serious gaming on orthopedic skills development. Each of these studies was published between 2016 to 2020, with two conducted in North America, and one each in Europe and South America. Of those who participated, 30 were laypeople, 30 were medical students, and 152 were orthopedic surgery residents.

**Table 1:** Characteristics of included studies. ABOS: American Board of Orthopaedic Surgery; OITE: Orthopaedic In-Training Exam; USMLE: United States Medical Licensing Examination

Author(s)	Year	Country	Study population (sample size)	Intervention	Assessment	Results
Jentzsch et al. [23]	2016	Switzerland	Laypeople (30)	Video game (Connect Four, Tetris, Fifa 2014, Rayman Legends, Call of Duty)	Time to perform the task successfully, cartilage damage to the femur and tibia, overall intra-articular distances of the camera, hoo. and punch (triangulation) during each task, as well as the removed (pathologic and healthy) meniscus	Experienced and good 3D gamers performed better than non-experienced and poor 3D gamers on the arthroscopy simulator
Egol et al. [25]	2017	USA	Current and former residents (120)	Video game (Wasabi Waiter Video game)	Performance on Step 1 of USMLE, yearly OITE scores, passage of Part I of the ABOS certifying examination, and clinical skills ratings compiled from rotation evaluations during their training	Video game dynamics can measure behavioral markers and help identify characteristics that may predict success during orthopedic surgery residency
Gonzalez et al. [27]	2018	Peru	Medical students (30)	HTC Vive Immersive Virtual Reality Headset	Results of the game, including score, time, precision, errors, and sequence	Helped students improve their medical skills and motivated them to improve for actual surgery
Blevins et al. [28]	2020	USA	Residents (32)	Competitive Environment with Scores	Timed simulated tasks: cadaveric carpal tunnel release, sawbones model of total knee arthroplasty, sawbones model of ankle fracture open reduction internal fixation, and knee arthroscopy simulator	Residents found the exercises to be valuable to their surgical education

Game Characteristics

Two of the four studies focused on the use of video games, spanning from classic games such as Tetris and Connect Four to more advanced role-playing or first-person shooter games such as Wasabi Waiter and Call of Duty. One study utilized a virtual reality simulator headset for participants to complete a task related to cruciate ligament surgery of the knee. The last study simulated a competitive Olympic-styled environment by scoring the participants on each station-based orthopedic surgery-related task.

Study Themes

Two studies utilized training simulators to assess surgical skills objectively, one study used video gaming to identify behavioral markers and resident characteristics to predict success within a training program, and the last study used a competition-based amalgamation of cadaveric, sawbones-based, and arthroscopic tasks to evaluate resident training progress.

Three studies showed a positive correlation between video gaming experience and orthopedic skill performance, acquisition, or both. The last study showed a positive response from residents when training sessions were hosted in a competitive, but friendly environment with direct observation from their attendings.

Discussion

This systematic review analyzes the studies conducted on gamification and the utilization of serious games in orthopedic surgery education. Unlike the games “Underground” and “Super Monkey Ball,” which have been extensively validated as training tools for laparoscopic motor skills, the development of serious games in orthopedic surgery has not been well-explored [4]. The games involved in the studies included in this review are not validated; hence, any positive correlations between gamification and improved performance for specific clinical skills may be imprecise. Well-constructed and validated games will be necessary to prove the potential benefits of gamified learning in orthopedic surgery education.

Current Studies of Gamification and Serious Games in Orthopedic Surgery

When comparing gamified learning to traditional orthopedic residency training, there are several unique benefits to note. Jentzsch et al. showed a positive correlation (r = 0.50; p = 0.005) between cumulative video game experience and performance during knee arthroscopy simulation. Additionally, three-dimensional (3D) game performance showed a positive correlation with tasks such as triangulation and meniscus resection on the simulator (both r ≥ 0.60; p < 0.001). Given that this study was conducted with non-medicine adjacent participants, it illuminates the benefits of exposure to fast-paced, aim-based games in the acquisition and training of specific orthopedic surgical skills, in addition to cognitive training, expanding learning capabilities, and perceptual development, all of which can vitally enhance performance in orthopedics [23].

The competition was identified as an important motivator in the improved performance of medical students and residents by both Gonzalez et al. and Blevins et al [27,28]. This theme is common among extant studies that evaluate the relationship between competition and motivation or performance [29-31]. Simulating a competitive but friendly environment can stimulate active learning and higher rates of satisfaction [29]. Gonzalez et al. explored the use of a fully immersive virtual reality headset in orthopedic surgery simulation training using the HTC Vive headset (HTC Corporation, New Taipei, Taiwan) with medical students as subjects, with an objective performance metric. The 30 participants were each given two attempts, one with a tutorial and one without, with everyone performing more proficiently in their second attempt. The study claims that while the students found the experience enjoyable, the main reason for the significant difference in scores between attempts was their determination to improve their scores [29]. Similarly, Blevins et al. hosted an Olympic-styled competition that saw orthopedic surgery residents exhibit their skills in four orthopedic disciplines: a cadaveric carpal tunnel release, a total knee arthroplasty on a Sawbonexâ model, an open reduction and internal fixation of an ankle fracture on a Sawboneâ model, and a knee arthroscopy simulation [28]. Their performance was scored on a standardized evaluation sheet by a fellowship-trained attending surgeon. In addition to the competitive environment eliciting drive and the determination to perform well within the residents’ own training batch, the competition provided an opportunity to improve orthopedic skills through direct observation and feedback from their attendings.

Development and Future Directions

Aside from technical improvements, gamified learning has shown numerous benefits for the improvement of training curricula in general. Creating an environment for friendly competition with point-based reward systems improves overall engagement. Gamified learning increases attentive participation and motivated learning via incentive-based surgical education [27,28]. There are many opportunities to implement this style of learning within a notably tedious and often lecture-based field of study. Though this paper has primarily discussed the use of video games and 3D simulation, gamified learning can be established through financially viable methods. Simply adding an incentive-based learning method that simulates a competitive environment can increase engagement at any level of learning. One example of this would be to implement a Jeopardy-style trivia competition as a bonding activity between residents of different years of training to assess clinical knowledge. The cumulation of points with a reward-based incentive for performance should, in theory, increase engagement in preparation and active learning. When comparing this style of gamified learning to the acquisition of 3D simulators, it becomes evident that there remain multiple opportunities for gamified learning that appeal to all educational budgets.

To date, there are limited gamified options that have been specifically developed to address the educational gap in orthopedic training. While “Underground” (Cutting Edge B.V., Leeuwarden, The Netherlands) is a validated serious game meant to help train residents in skills pertinent to MIS, the majority of injuries that are treated by orthopedic surgeons cannot be addressed arthroscopically. Hence, there remains a need for validated and cost-effective solutions for other orthopedic skills. Nguyen et al. and Gani et al. investigated the role of low-end haptic devices which can create realistic experiences with forces and vibrations as a solution in simulating bone drilling [26,32]. This can obviate the requirement of using disposable sawbones in training. Additionally, the use of haptic devices in virtual reality can present the opportunity to create unique clinical scenarios that homogenous sawbones cannot. Different patterns of the same or similar injury that are more arduous to address can correspond with the increasing difficulty of each “level,” allowing for the trainee to experience diverse clinical scenarios they may encounter and adequately prepare them for operations. These scenarios in virtual reality can also be repeated indefinitely without the use of expendable materials until the user is comfortable with a certain technique or approach, proving another advantage of this technology. This is just one of the many possibilities in the relatively unexplored realm of orthopedic residency education, and this review has illuminated the need for funding to further develop other training methods to uncover the full potential of gamified learning.

Limitations

While the authors believe that all relevant articles were captured by the search strategy and selected precisely with the filtration process, only four articles were included in this systematic review. This is in comparison to the numerous studies on gamification and serious games in other fields such as gastroenterology or general surgery. Although the skill sets for endoscopic, laparoscopic, and arthroscopy procedures are similar, the purpose of this review was to elucidate the current use of gamification and serious games in orthopedic surgery. In addition, the quality of each study was difficult to address due to the methodological differences and lack of validation, as well as the wide variety of game genres each participant gravitated towards before the study based on their own preferences. As such, future studies should differentiate between participants by the genre of games they are most experienced with, such as first-person shooter or sandbox games, as the skills acquired through different genres may produce different results. Nonetheless, this summary of studies provides an adequate basis for the future exploration of applications using serious games or other gamification elements to orthopedic residency training.

## Conclusions

As the field of medicine rapidly progresses, so will residency education and training. The implementation of gamified learning has the potential to address notable gaps in traditional medical education, which is currently highly reliant on notetaking, self-motivated repetition, and formal examinations with the absence of progress tracking. Serious games can provide extrinsic motivation and instantaneous feedback to trainees, as well as direct goal-oriented tracking of content learning. Similarly, gamified learning in residency training can allow for mistakes and flaws in a low-stake setting which can often lead to improvement, especially in skills with a notable learning curve. Gamified learning has shown numerous potential benefits and can be implemented flexibly in institutions at various levels based on their individual and unique requirements. There remain vast potential impacts of gamified learning in orthopedic surgery education, including the potential to hone problem-solving skills, refine technical and non-technical skills, and improve the efficiency of learning.

## Appendices

Appendix 1: Search strategy

Ovid MEDLINE (n = 265)

1.     gam*.mp or video gam*.mp. or gamif*.mp. or serious gam*.mp or Wii.mp or console.mp

2.     exp medical education/ or exp surgical education/ or resident*.mp or intern*.mp or medical student?.mp or train*.mp

3.     exp surgery/ or surgical skill?.mp or orthop?edic*.mp or arthroscop*.mp

4.     1 and 2 and 3

5.     limit 4 to english language

6.     limit 5 to yr= “2012 - Current”

Web of Science (n = 1,785)

1.     gam* or video gam* or gamif* or serious gam* or Wii or console*

2.     medical education/ or surgical education/ or resident* or intern or internship or medical student? or train*

3.     surgery or surgical skill? or orthop?edic* or arthroscop*

4.     2012-01-01 to 2023-07-01

Scopus (n = 310)

1.     "gam*" OR "video gam*" OR "gamif*" OR "serious gam*" OR {Wii} OR {console}

2.     "medical education" OR "surgical education" OR "resident*" OR {intern} OR {internship} OR "medical student?" OR "train*"

3.     "surgery" OR "surgical skill?" OR "orthop?edic*" OR "arthroscop*"

4.     2012-2023
